# Acute Respiratory Distress Syndrome Induced by Parathyroid Storm

**DOI:** 10.7759/cureus.12881

**Published:** 2021-01-24

**Authors:** Georgios Zagkotsis, Maria Markou, Panagiota Papanikolaou, Nikolaos Sabanis

**Affiliations:** 1 Nephrology, General Hospital of Livadeia, Livadeia, GRC; 2 Endocrinology, “Evaggelismos” General Hospital of Athens, Athens, GRC

**Keywords:** primary hyperparathyroidism, parathyroid storm, hypercalcemia, metastatic calcifications, acute respiratory distress syndrome, chronic kidney disease, hemodialysis, calciphylaxis

## Abstract

Hypercalcemic crisis associated with the development of acute respiratory distress syndrome (ARDS) has been rarely documented in the literature. Most cases have been described in patients suffering from malignancies or renal failure with the presence of metastatic calcifications being a prominent feature. Only three cases of ARDS have been reported to date in patients with hypercalcemic crisis due to primary hyperparathyroidism (PHPT). Herein, we report a 72-year-old patient with PHPT that presented with severe hypercalcemic crisis and developed ARDS. He had mild chronic kidney disease and at presentation he had extremely high levels of serum calcium (22.5 mg/dl) and parathormone (3822 pg/ml). After receiving medical treatment for hypercalcemia and the initiation of hemodialysis, he developed ARDS with a fatal outcome, without the presence of pancreatitis, sepsis or heart failure. Although very rare, ARDS should be taken into account as a possible complication of parathyroid crisis, especially in patients with excessive calcium and parathormone levels.

## Introduction

The terms parathyroid storm and parathyroid crisis have been used to describe hypercalcemic crisis that occurs in the course of primary hyperparathyroidism (PHPT) [1]. It is an uncommon but potentially life-threatening complication of PHPT that, even though it has no uniform standard definition, is usually recognized by an excessively elevated serum calcium (sCa) level, greater than 14 mg/dl, along with rapid deterioration of central nervous system, cardiovascular, gastrointestinal, and renal functions. The development of cardiac arrhythmias and pancreatitis is associated with increased mortality [2, 3]. Acute respiratory distress syndrome (ARDS) has been described in patients with hypercalcemic crisis associated with malignancies [4], but only two cases of ARDS related to parathyroid crisis have been reported since 1974 [5, 6]. We report a patient that presented with parathyroid storm and developed ARDS without the presence of pancreatitis or decompensated heart failure.

## Case presentation

**Figure 1:**
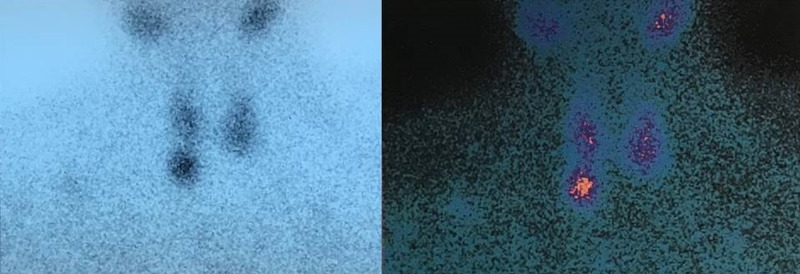
Tc 99m-Sestamibi scintigraphy indicative of parathyroid adenoma below right inferior parathyroid lobe.

A 72-year-old Caucasian male patient presented in the emergency department of our district hospital with generalized weakness, vomiting, altered mental status and confusion over the last four days. He had a history of arterial hypertension, mild chronic kidney disease (serum Cr: 2.0 mg/dl), coronary artery disease with coronary artery bypass grafting and permanent pacemaker implantation four years ago. Six months prior to his admission he was found to have sCa of 12.1 mg/dl, serum phosphorus of 1.9 mg/dl and parathormone (iPTH) of 685 pg/ml and he was diagnosed with PHPT. Tc 99m-Sestamibi scintigraphy detected an area of increased capture of radiotracer located at the right inferior thyroid lobe, indicating parathyroid adenoma (Figure 1).

Although it was recommended, the patient refused to undergo parathyroidectomy and he was on cinacalcet 60 mg per day, but his relatives reported him being incompliant with treatment. He was also on aspirin (100 mg per day), bisoprolol (5 mg per day), furosemide (20 mg per day) and allopurinol (100 mg per day).

On admission he was lethargic and dehydrated, but he was hemodynamically stable (BP: 130/70 mmHg, HR: 80 bpm) with SaO2 98% and afebrile. There were no significant findings on clinical examination, chest X-ray, abdominal ultrasound and CT scan. Blood tests revealed an extremely high sCa of 22.5 mg/dl and an iPTH of 3822 pg/ml. He had increased white cell count (12.5 x 10^9^/L) with a C-reactive protein (CRP) of 20.5 mg/dl. His amylase was slightly raised (190 U/L), total bilirubin was 2.5 mg/dl (direct bil: 1.6 mg/dl), lactate dehydrogenase (LDH) was 280 U/L and both transaminases were at the level of 50 U/L. He had sodium of 137 mmol/l, potassium of 3.7 mmol/l and magnesium of 2.1 mg/dl, but his phosphorus was 5.4 mg/dl resulting in extremely high calcium X phosphorus product (121). His renal function was mildly deteriorated compared to baseline (sCr: 2.5 mg/dl, Urea: 146 mg/dl), his pH was 7.35 with bicarbonate of 21 mmol/l.

Intravenous fluids were administered at a rate of 250 ml/hr, broad spectrum antibiotics were commenced (ciprofloxacin and metronidazole) and he was given a single dose of zoledronic acid 2 mg. A central venous catheter was placed and he underwent two subsequent hemodialysis sessions. After the first 24 hours patient’s clinical status improved. He was alert (GCS: 15/15), hemodynamically stable with SaO2 96% on room air, he had a urine output of 4 litres/day, his sCa decreased at the level of 14.5 mg/dl after the second hemodialysis session and his CRP to 9.8 mg/dl.

**Figure 2:**
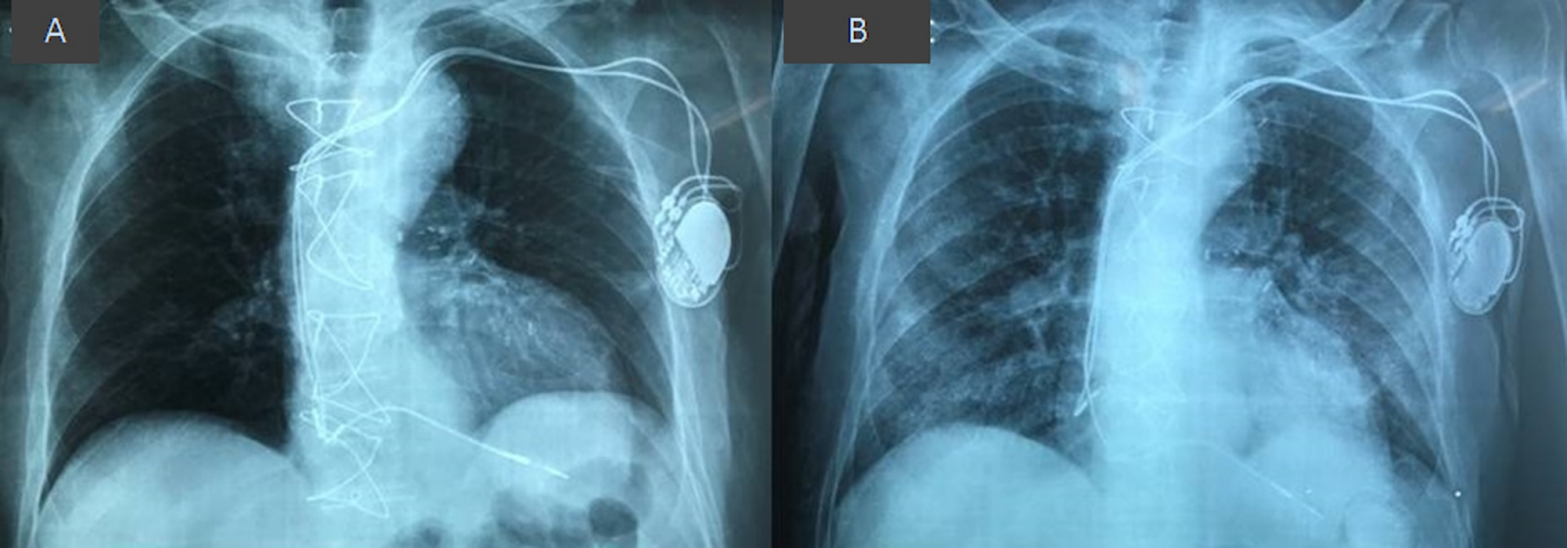
(A) Chest X-ray on admission. (B) Bilateral pulmonary infiltrates on chest X-ray 24 hours later.

During the second day, the patient developed respiratory distress with SaO2 82% on room air, but he was hemodynamically stable. On auscultation, bilateral fine rales were found all over the lungs. Chest X-ray demonstrated bilateral widespread pulmonary infiltrates (Figure 2).

**Figure 3:**
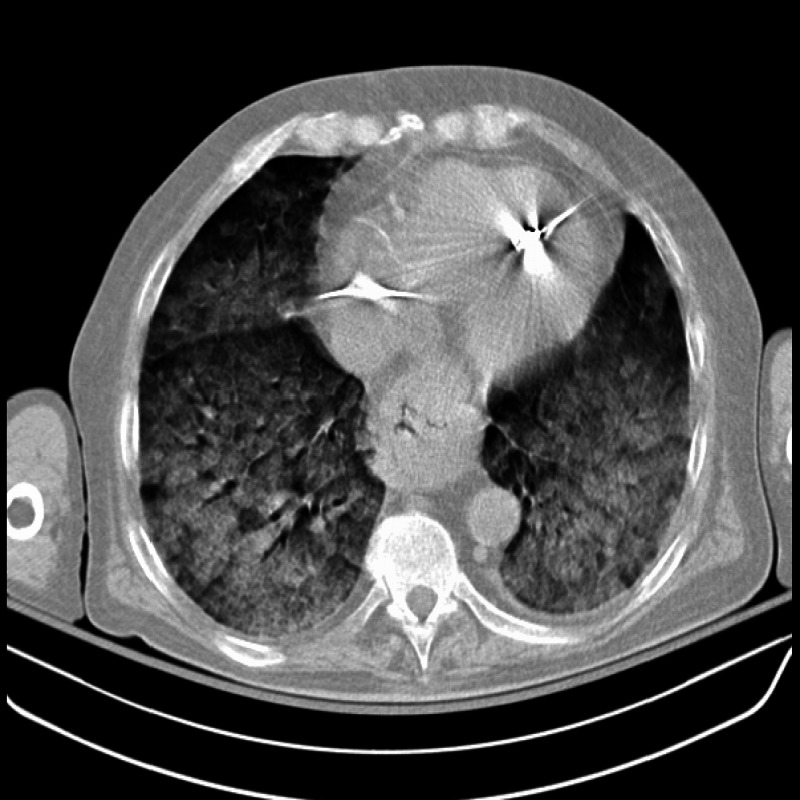
Chest CT scan indicative of severe ARDS. ARDS: Acute respiratory distress syndrome

CT scan revealed diffuse pulmonary opacifications bilaterally, indicating severe ARDS (Figure 3).

Cardiogenic pulmonary oedema was unlikely, as jugular vein pressure was very low (4 cm H2O), while ejection fraction was 50% on heart ultrasound with no signs of decompensated heart failure. The patient received high flow oxygen, hydrocortisone, meropenem, vancomycin and amikacin. Blood cultures were obtained, but they were negative. His condition deteriorated the next couple of hours and he required continuous infusion of vasopressors. He was intubated and mechanical ventilation was initiated, but he died before reaching the local intensive care unit.

## Discussion

ARDS is a very rare complication of hypercalcemic crisis, especially in the clinical course of PHPT, since most cases are associated with malignancies [4, 7, 8]. Holmes et al. were the first to describe a case of ARDS in a patient with parathyroid crisis in 1974 [5]. Surprisingly, almost 40 years had to pass for the second case to be reported by Mert et al. in 2012 [6]. Recently, Cummings et al. described another patient that developed multi-organ failure due to PHPT-related hypercalcemia, affecting respiratory, renal and cardiovascular systems [9]. These patients, as the one we present, had not developed pancreatitis, a potentially devastating complication of hypercalcemic crisis that may lead to multi-organ failure, hence ARDS [7, 10]. Apart from pulmonary infections and sepsis, cardiogenic pulmonary edema should always be excluded, as hypercalcemia may trigger cardiac arrhythmias and accelerate vascular and valvular calcification. PTH acts on cardiomyocytes independently of sCa levels promoting left ventricular hypertrophy and fibrosis that lead to diastolic dysfunction and suppressed contractility [3, 11]. These effects have been mainly described in patients with chronic kidney disease and long lasting hyperparathyroidism [12]. In the case reported by Cummings et al., high output heart failure may have resulted or at least contributed in the development of pulmonary edema [9]. On the other hand, the patient we report had chronic kidney disease and extremely high levels of PTH and sCa, but the diagnosis of cardiogenic pulmonary edema was unlikely. Despite the fact that right heart catheterization with a Swan-Ganz catheter was not performed in order to definitely exclude cardiogenic pulmonary edema, his central venous pressure was low (4 cm H2O) and heart ultrasound indicated no signs of decompensated heart failure.

The pathophysiological mechanism involved in the induction of acute lung injury in hypercalcemic crisis remains elusive. In the case reported by Holmes et al., autopsy revealed extensive calcium deposition in the alveolar cells and the authors suggested that probably this depositions destroyed alveolar/capillary barrier leading to lung injury [5]. Likewise, in a case series of malignant hypercalcemia reported by Hsu and Chen, autopsies were also performed and metastatic calcifications were present in the lungs as well as other organs like stomach, heart and kidneys [4]. In the former reports, an extremely high sCa level, above 20 mg/dl, appeared to be a risk factor for developing lung injury [4, 5]. On the other hand, in the case presented by Mert et al., lowering sCa levels towards normal led to significant improvement of respiratory failure, although this patient did not have initially extremely high sCa levels [6]. An experimental study in conscious rats and isolated perfused rat’s lungs, performed by Chen et al., suggested that hypercalcemia may produce a sepsis-like syndrome and acute lung injury by increasing plasma nitrate/nitrite, free radicals, proinflammatory cytokines, procalcitonin and iNOS activity [13].

It is also speculated that a high calcium-phosphorus product may lead to enhanced precipitation of calcium in relatively alkaline areas like alveolar capillaries and venules [5]. The presence of renal failure seems to promote calcium deposition and metastatic calcifications eventually consist a predominant characteristic of patients with end stage renal disease (ESRD) or acute renal failure [14, 15]. It has been also suggested that initiation of dialysis may precipitate calcification by reversing a low systemic pH towards alkaline [14]. The patient we report had chronic kidney disease with only mild deterioration of renal function at presentation, but not ESRD or severe acute renal failure. ARDS occurred after initiation of dialysis and the possibility that dialysis triggered ARDS should be taken into account, since his calcium-phosphorus product was over 100, despite not being profoundly acidotic.

A devastating form of calcification may occur in small and medium vessels’ walls, accompanied by intimal proliferation, fibrosis and necrosis. This is a potentially fatal condition that leads to tissue ischemia and necrosis, referred as calcific uremic arteriolopathy (CUA) or calciphylaxis [16]. Calciphylaxis and metastatic calcifications are probably manifestations of the same process that share the same predisposing factors like hypercalcemia, high calcium-phosphorus product and high levels of proinflammatory cytokines. Their main histopathological difference is the presence of ischemic and necrotic lesions in tissue biopsies [14, 17]. CUA is mainly, but not exclusively, encountered in ESRD patients, since it has been reported in several conditions including PHPT and malignancies [18]. It is regarded as a systemic disease but, almost always, skin, subcutaneous fat or muscles of extremities are involved resulting in ulcerations. Nonetheless, visceral calciphylaxis has been described with diffuse tissue calcification which affects lungs, myocardium, kidneys or even pancreas [14, 17, 19]. Regarding our patient, the development of CUA cannot be excluded, since tissue biopsies were not obtained and autopsy was not performed. However, pulmonary calciphylaxis is probably unlikely since ulcerating skin lesions were not observed and imaging studies did not reveal calcifications in any organ.

It is worth mentioning a single case report by Trivedi et al., in which the administration of zolendronic acid was followed by systemic inflammatory response syndrome (SIRS), shock, ARDS and end-organ dysfunction. The authors speculate that the elevation of inflammatory cytokines after zolendronate infusion may promote the development of SIRS. However, there are significant differences between this case and the one we present. This was a 7-year-old pediatric patient with complex medical problems like quadriplegic cerebral palsy and chronic ventilation support who had been submitted to several surgical procedures the previous day. These conditions may have predisposed to the development of SIRS. He did not have hypercalcemia or hyperparathyroidism and zolendronate was given for osteoporosis secondary to immobilization and vertebral compression fractures. SIRS was initiated only 3 hours after zolendronic acid infusion leading to shock and ARDS within 12 hours. On the contrary, our patient developed ARDS almost 36 hours later, by the time he had completed his second dialysis session. Moreover, this pediatric patient developed complications not seen in our patient, like leucopenia, thrombocytopenia, coagulopathy and extreme elevation of CRP without acute kidney injury or deterioration of renal function [20].

## Conclusions

In conclusion, ARDS is a very rare complication of hypercalcemic crisis secondary to PHPT. Most cases of hypercalcemia-associated pulmonary edema have been described in patients with malignancies. Given the cardiotoxic effects of hypercalcemia and parathormone, cardiogenic pulmonary edema should be taken into account in patients with parathyroid crisis, while pancreatitis and sepsis should be thoroughly investigated. Serum calcium levels above 20 mg/dl appear to be a major factor for the development of ARDS. Autopsy studies suggest that calcium deposition in alveolar epithelial and endothelial cells may cause lung injury. Hence, pulmonary edema is more often encountered in patients with metastatic calcifications in the lungs or other organs. It is possible that parathyroid storm may cause a form of acute and rapid lung calcification leading to ARDS. Renal failure and especially ESRD is a major predisposing factor for calcium precipitation and the initiation of dialysis may enhance this process by altering pH, especially in patients with high calcium-phosphorus product.
